# The Use of Wearable Inertial Motion Sensors in Human Lower Limb Biomechanics Studies: A Systematic Review

**DOI:** 10.3390/s101211556

**Published:** 2010-12-16

**Authors:** Daniel Tik-Pui Fong, Yue-Yan Chan

**Affiliations:** 1 Department of Orthopaedics and Traumatology, Prince of Wales Hospital, Faculty of Medicine, The Chinese University of Hong Kong, Hong Kong, China; 2 The Hong Kong Jockey Club Sports Medicine and Health Sciences Centre, Faculty of Medicine, The Chinese University of Hong Kong, Hong Kong, China; 3 Department of Orthopaedics and Traumatology, Alice Ho Miu Ling Nethersole Hospital, Hong Kong, China

**Keywords:** inertial sensors, accelerometers, gyroscopes, magnetic sensors, joint kinematics, motion analysis

## Abstract

Wearable motion sensors consisting of accelerometers, gyroscopes and magnetic sensors are readily available nowadays. The small size and low production costs of motion sensors make them a very good tool for human motions analysis. However, data processing and accuracy of the collected data are important issues for research purposes. In this paper, we aim to review the literature related to usage of inertial sensors in human lower limb biomechanics studies. A systematic search was done in the following search engines: ISI Web of Knowledge, Medline, SportDiscus and IEEE Xplore. Thirty nine full papers and conference abstracts with related topics were included in this review. The type of sensor involved, data collection methods, study design, validation methods and its applications were reviewed.

## 1. Introduction

Wearable inertial motion sensors consisting of accelerometers, gyroscopes and magnetic sensors are readily available nowadays [1]. Some companies, such as XSens Technologies (The Netherlands) and Innalabs (Russia) provide inertial motion sensor solutions. They are highly transportable, no stationary units, such as receivers and cameras are needed for data collection, therefore can be used outside laboratory conditions [2]. Inertial motion sensor is a good choice for human biomechanics studies because it is highly transportable, low cost and consumes low power during operation.

Accelerometers have been adopted in human joint kinematics studies since 1990s. Willemsen [3] and Heyn [4] applied uniaxial accelerometers on aluminum strips, which were then attached on the foot, shank, thigh and pelvis of subjects by Velcro straps. In their studies, four accelerometers on rigid metal plates were needed on each segment, otherwise they would have had to numerically integrate twice the angular acceleration of the segment to get the joint angle [5]. Therefore a total of eight accelerometers were needed to estimate joint kinematics. Only uniaxial joint kinematics could be obtained. Also, in both studies, leg segments were assumed to be rigid bodies, and the joints were single axis hinge joint. These simplified joint models were good for simple motion analysis, for example two dimensional single joint motion analysis.

Simplified systems were developed in 2000s. Data from accelerometers and gyroscope could be used to estimate orientation relative to an inertial frame [1]. Although relative orientation could be estimated by integration of data from gyroscope, errors would accumulate by this method, which caused distortion and drift errors. Accelerometer can be used to compensate the drift of the gyroscope about the axes of the horizontal plane, while magnetic sensor which located orientation by earth’s magnetic field was adopted to solve this drift problem about the vertical axis [6]. However, inside reinforced-concrete-covered buildings, the magnetic field on the earth was always perturbated. Further development of high accuracy three dimensional relative orientations was developed by Favre and his colleagues [7]. Favre and his colleagues integrated angular velocity data obtained from gyroscopes, and then corrected the angle estimation based on inclination data from accelerometers gathered during rest or constant velocity motion period. Known joint anatomical constraints were also considered for better estimation in a later study [8]. Static calibration in a defined position was still needed. Cooper [1] and his colleagues extended the measurements in dynamic activities. However, Cooper’s studies only involved a simplified model of a single hinge knee joint, further extension of the technique was needed for three dimensional measurements.

## 2. Methods

**Figure 1.**
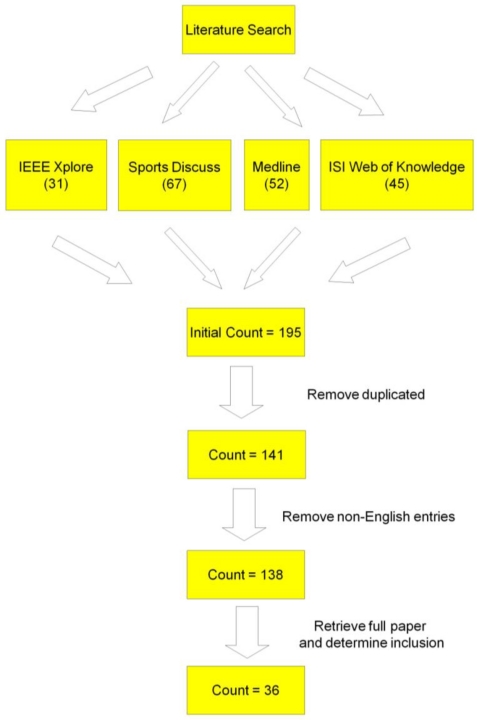
Research method of this study.

The research method was graphically displayed in Figure 1 for better understanding of the procedure. Systematic literature search of Medline (from 1966), ISI Web of Knowledge (Science Citation Index Expanded, from 1985; Social Sciences Citation Index, from 1956; Arts & Humanities Citation Index, from 1975), SportDiscus (from 1975) and IEEE Xplore was conducted at the last week of July in year 2010. The four databases were chosen as they were popular search engines which cover most of the literature in engineering, medicine and sports biomechanics field. The searched keyword string was “(biomechanics OR injury prevention OR kinematics) AND (lower limb OR knee OR hip OR ankle) AND (inertial sensor OR accelerometer OR gyroscope OR gyrometer OR magnetic sensor OR magnetrometer)” appeared in title, abstract, and keyword fields. The initial total number of identified articles from these databases was 195. Fifty four duplicated entries were moved, therefore 141 articles were left. Three articles not written in English were excluded, the number of articles were further reduced to 138. These 138 full papers were obtained from the library in The Chinese University of Hong Kong as well as from online search. The title and abstract of each entry was read, non-related studies were excluded, 36 full papers and conference abstract were left. Three more papers in related topic were added manually [9–11], therefore, a total of 39 full papers and conference abstract were included in this review. Inclusion criteria were as follow: (1) The study reported lower limb joint kinematics; and (2) The study involves accelerometers, gyroscopes and/or magnetic sensors. However, articles only contain the following content were excluded: (1) *In vivo* and *in vitro* kinematics studies; (2) Joint kinematics data not obtained from accelerometers, gyroscopes or magnetic sensors and (3) Frequency analysis.

## 3. Results and Discussion

### 3.1. Type of Sensors

Type of sensors used ranged from uniaxial accelerometers to triaxial accelerometers, gyroscopes and magnetic sensor. Full scale of accelerometers ranged from 3 g to 10 g, those of gyroscope ranged from 300–1,200 degree/second. For magnetic sensors, the full scale was 750 mGauss [12,13]. The weight of motion sensors adopted ranged from 18.2 g to 700 g, and the size ranged from 20 × 10 × 7.2 mm^3^ to 64 × 62 × 26 mm^3^. Sampling frequencies of these systems ranged from 20–800 Hz. Details of sensors used in the studies were shown in Table 1.

### 3.2. Data Logging and Processing

In most of the reviewed papers, collected data was not processed in a real time basis. Some of the systems have its own data logging system attached on the subject’s body. Portable data loggers with different types of memory cards, for example, flash memories and SD-micro cards [1,8,16,20,25,27,31] were one of the common methods for data logging. These memory cards allow handy data storage. There were also systems which required subjects to carry a hand held PC with them for data collection [34]. Hand held PC allows data collection in daily activities as they can be carried in pocket easily. However, their sizes were still not small enough for subjects performing vigorous sport motions. Wired systems which data were collected by a wired notebook PC also existed [17,30]. Bluetooth wireless communication was also adopted [9,33], which allows subjects to have more freedom of motion during data collection. However, workstation must present for data collection, therefore it is not suitable for ambulatory system.

One of the disadvantages of application of wearable sensors in human motion analysis was that noise in data collection was usually severe. Therefore, data have to be filtered before further processing. Low pass filters with cut off frequencies ranged from 15–40 Hz were adopted in various systems [2,3,6,26,29]. The cut off frequencies were chosen carefully based on the motions being performed. Butterworth filters [17,26], Kalman filters [1,10,37] and Savitzky-Golay filters [18,19] were also adopted in some other systems according to their applications and motions to be detected (Table 2). Curved fitting technique was also adopted to eliminate noise [33].

### 3.3. Study Design and Validation

Most of the studies reviewed recruited young (age 18–40) healthy individuals as subjects. Only two studies have older subjects with average age of aged 58.7 [18,19]. Sample size ranged from one to 36. Walking and running on flat ground or treadmill were common motions being analyzed [1,2,4,8,14,16–18,21,22,24,27,29,31,34,36,37,39]. However, some other specific motions were also involved, for example, walking on difference surfaces [26], stand-sit transition [34], landing from a fall [15], tennis serve [38], rowing, cycling [20], jumping, walking downstairs, cutting, simulated sprain [17], walking upstairs [2], knee and ankle joint movement [6,11,30].

The accuracies of the motion sensing systems were mostly compared with those of video cameras or high speed optical motion analysis systems with reflexive markers, as video cameras and optical motion analysis systems were commonly used for human joint kinematics assessment nowadays.

### 3.4. Applications

The reviewed papers monitored the joint kinematics of ankles, knees and hips. Most of the studies simplified these joints as simple hinge joints, which assume only sagittal plane movement was allowed. However, some studies could provide detailed three dimensional descriptions for ankle, knee and hip joints. Tibial acceleration was other commonly recorded parameters for human motion analysis. Tibial acceleration can be easily obtained from accelerometer data, without complicated data processing, therefore was favorable for real time monitoring and classification of different human activities.

By analysis of lower limb joint kinematics, several applications could be done, for example: Analysis of skill level and locomotor performance of athletes or patients [24,27,38]; ambulatory measurement to monitor patients’ daily activities [7,8,16,29]; clinical assessment for patients [13,30]; Gait event detection and analysis [1,18,22,23,26,37,39] and identification of different daily activities, for example stair climbing, walking, running, rowing, cycling and simulated ankle sprain [2,17,20].

### 3.5. Fixation Methods

Fixation methods were a very important part in motion analysis using motion sensors. A good fixation method reduced the artifices of skin movement. Moreover, good fixation methods can greatly reduced the errors caused by improper alignment to anatomical axes. Velcro straps [3,30], double-side adhesive tape [39], elastic straps [2,29,18,28] and neroprene straps [11] were commonly used for fixing motion sensors on subjects’ bodies [3,15]. These tapes and straps are flexible and convenience to use. However, errors caused by skin movement can be significant. Some of the sensors were fixed on aluminum plate [4,15,29,31,39] or put inside plastic casing [6,25] first before attaching on subject’s bodies. Hard plate and casing can reduce relative skin movement and protect the sensors from damage. However, they are usually heavy and restrict subjects from normal movement. Semi-rigid belt [27] and exoskeleton [21] harness were also used for better sensor attachment, but are not convenience for long term ambulatory use. Another important note for fixation of sensor was that we have to ensure the axes of the inertial sensors aligned with the anatomical axes of the segments. Some studies applied anatomical calibration to align sensor’s axes with the axes of the body segment. Calibration devices were adopted in static trial to identify the lines connecting anatomical landmarks [32]. Static postures were also adopted to calibrate the sensors in a functional approach [9].

### 3.6. Discussion

Some limitations still exist for lower limb human biomechanics analysis by wearable inertial motion sensors. Firstly, filtering, integration, trigonometry were involved to estimate joint angular kinematics. Therefore, high demand of hardware was needed for data processing, this made a higher cost and larger size for the data processing unit. Most importantly, complicated data processing technique means real time analysis nearly impossible in most of the studies. In some other studies, real time analysis was possible, for example Cutti [9] and his colleagues can measure real time joint kinematics, however, it was still limited to data collection in clinical setting.

Future development of joint kinematics analysis techniques for wearable inertial sensors should focus simplification of data processing algorithm would be the most challenging part. Batteries lives, fixation method, size of central processing unit were other aspects to be improved. Inertial sensors might replace video cameras and optical motion analysis systems in some human biomechanics studies as data collection could be done outside laboratory settings. The low cost of inertial sensors is obviously another advantage against optical motion analysis systems. Most importantly, it might be applied in ambulation system for real time motion classification, feedback to athletics about sports performance, monitor patients’ daily activities and even act as alarm for activation of protective mechanism when the user was in danger of sports injury.

## 4. Conclusions

Wearable inertial motion sensors are highly transportable and no stationary units, such as receivers and cameras are needed in data collection, therefore can be used outside laboratory conditions [2]. Due to the development of micro-electro-mechanical systems (MEMS), the size and power consumption were greatly improved in the design of sensor, making it a good choice for lower limb joint kinematics studies. However, data logging, data processing and fixation method are the areas to be improved in the near future. Simplify data processing algorithm can allow reduction of size and cost of the data processing unit, which allows easy attachment on users for ambulatory purpose. Fixation method which allows freedom of movement and minimizes skin movement is another important aspect.

## Figures and Tables

**Table 1.** Type of sensors adopted in reviewed studies.

**Source**	**Number of sensor module**	**Components of each sensor module**			**Sizes**	**Weight**	**Sampling frequency**
		**Accelerometer**	**Gyroscope**	**Magnetic Sensor**			
Cooper 2009 [1]	1	triaxial	triaxial				100 Hz
Coley 2005 [2]	1		uniaxial		30 × 30 × 30 mm^3^		
Willemsen 1991 [3]	16	uniaxial					500 Hz
Heyn 1996 [4]	8	uniaxial					100 Hz
O’Donovan 2007 [6]	2	triaxial	triaxial	triaxial	60 × 40 × 24 mm^3^		500 Hz
Favre 2008 [7]	2	triaxial	triaxial	triaxial	30 × 25 × 25 mm^3^		200 Hz
Favre 2006 [8]	2	triaxial	triaxial	triaxial			
Cutti [9]	10	triaxial	triaxial	triaxial	39 × 54 × 28 mm^3^	38 g	
Van den Noort 2009 [11]	2	triaxial	triaxial	triaxial			100 Hz
Kawano 2007 [12]	2	triaxial	triaxial	triaxial	53 × 38 × 21 mm^3^		100 Hz
Kawano 2008 [13]	2	triaxial	triaxial	triaxial	53 × 38 × 21 mm^3^	30 g	200 Hz
Zijlstra 2008 [14]	2	triaxial	triaxial		64 × 62 × 26 mm^3^	150 g	
Andrews 2000 [15]	1	uniaxial				18.2 g	
Avor 2009 [16]	4	triaxial	triaxial				
Chan 2010 [17]	1	triaxial	triaxial		20 × 18 × 6 mm^3^		500 Hz
Dejnabadi 2005 [18]	2	biaxial	uniaxial		20 × 20 × 10 mm^3^		200 Hz
Dejnabadi 2006 [19]	4	biaxial	uniaxial		20 × 20 × 10 mm^3^		
Ermes 2008 [20]	3	2 triaxial		1 triaxial			20 Hz
Favre 2009 [21]	2	triaxial	triaxial	triaxial			240 Hz
Findlow 2008 [22]	2	triaxial	triaxial		54 × 39 × 28 mm^3^		
Hanlon 2009 [23]	2	biaxial					200 Hz
Helot 2005 [24]	2	triaxial		triaxial			100 Hz
Kendell 2009 [25]	3	triaxial	triaxial	triaxial			
Lau 2009 [26]	2	biaxial	uniaxial		20 × 10 × 10 mm^3^		
L’Hemette 2008 [27]	1	triaxial				700 g	100 Hz
Liu 2008 [28]	2	triaxial					
Liu 2009 [29]	3	triaxial					
Mamizuka 2007 [30]	1	triaxial					
Mayagoitia 2002 [31]	8	uniaxial					100 Hz
Picemo [32]	4	triaxial	triaxial	triaxial			
Saber-Sheikh 2010 [33]	2	triaxial	triaxial	triaxial	53 × 38 × 21 mm^3^	30 g	
Simcox 2005 [34]	3	2 biaxial	1 uniaxial		70 × 50 × 25 mm^3^		800 Hz
Tong 1999 [35]	2		uniaxial		20 × 10 × 7.2 mm^3^		
Willemsen 1990 [36]	16	uniaxial					500 Hz
Zhang 2008 [37]	1	biaxial					
Ahmadi 2006 [38]	3	triaxial					500 Hz
Clark 2010 [39]	1	triaxial					

**Table 2.** Motions involved in reviewed studies.

**Source**	**Tested motions**
Cooper 2009 [1]	Walking at five speed from 1–5 mi/h
Coley 2005 [2]	Walking, stair climbing
Heyn 1996[4]	Walking
O’Donovan 2007 [6]	Heel and toe rise foot pumps, knee flexion and extension, clockwise and anti-clockwise ankle rotation, lateral and medial foot rotation, eversion and inversion, ambulation
Favre 2008 [7]	Knee abduction and adduction, 30 m flat walking
Favre 2006 [8]	Walking
Cutti 2010 [9]	Walking
Music 2008 [10]	Sit to stand movement
Van den Noort 2009 [11]	Clinical assessment of knee joint
Zijlstra 2008 [14]	Walking
Andrews 2000 [15]	Landing from a 5 cm fall
Avor 2009 [16]	Running on treadmill on three different speed
Chan 2010 [17]	Walking, running, jumping, walking downstairs, cutting, simulated ankle sprain
Dejnabadi 2005 [18]	Walking level at 3 km/h
Ermes 2008 [20]	Walking, running, rowing, cycling
Favre 2009 [21]	Walking
Findlow 2008 [22]	Walking at self-selected pace
Hanlon 2009 [23]	Walking
Helot 2005 [24]	Walking
Lau 2009 [26]	Walking level, upslope down slope, downstairs, upstairs
L’Hemette 2008[27]	Walking
Liu 2009 [29]	Walking at self-selected slow, normal and fast speeds
Mamizuka 2007 [30]	Knee flexion and extension
Mayagoitia 2002 [31]	Walking at 1.4 km/h, 2.1 km/h, 2.7 km/h, 3.6 km/h and 4.6 km/h
Picemo 2008 [32]	Walking
Saber-Sheikh 2010 [33]	Walking
Simcox 2005 [34]	Sit-stand-sit, walking
Willemsen 1990 [36]	Walking
Zhang 2008 [37]	Walking
Ahmadi 2006 [38]	Tennis serving
Clark 2010 [39]	Running on treadmill (10 km/h)
